# Gut-Derived Metabolites and Their Role in Immune Dysfunction in Chronic Kidney Disease

**DOI:** 10.3390/toxins12040245

**Published:** 2020-04-11

**Authors:** Griet Glorieux, Tessa Gryp, Alessandra Perna

**Affiliations:** 1 Nephrology Division, Ghent University Hospital and Ghent University, 9000 Ghent, Belgium; tessa.gryp@ugent.be; 2 First Division of Nephrology, Department of Translational Medical Sciences, School of Medicine, University of Campania “Luigi Vanvitelli”, 80131 Naples, Italy; alessandra.perna@unicampania.it

**Keywords:** uremic toxins, gut, chronic kidney disease, immune

## Abstract

Several of the uremic toxins, which are difficult to remove by dialysis, originate from the gut bacterial metabolism. This opens opportunities for novel targets trying to decrease circulating levels of these toxins and their pathophysiological effects. The current review focuses on immunomodulatory effects of these toxins both at their side of origin and in the circulation. In the gut end products of the bacterial metabolism such as p-cresol, trimethylamine and H_2_S affect the intestinal barrier structure and function while in the circulation the related uremic toxins stimulate cells of the immune system. Both conditions contribute to the pro-inflammatory status of patients with chronic kidney disease (CKD). Generation and/or absorption of these toxin precursors could be targeted to decrease plasma levels of their respective uremic toxins and to reduce micro-inflammation in CKD.

## 1. Introduction

Under physiological conditions, cells and tissues are maintained in a homeostatic state while under pathophysiological conditions they experience stress. The latter conditions require adaptation in which the immune system plays an important role. The characteristics of this adaptive response are an intermediate between the basal homeostatic state and an inflammatory response, called low-grade inflammation, switched on in order to restore functionality and homeostasis [1]. Inflammation is mediated by an interaction of multiple components of the innate and adaptive immune system including complement factors, leukocytes and cytokines. Chronic kidney disease (CKD) is a state of chronic, low-grade inflammation which contributes to the accelerated progression of chronic inflammatory disturbances affecting immune balance with increased levels of innate immunity biomarkers, such as C-reactive protein (CRP) and interleukin (IL)-6. Causes and consequences of inflammation and CKD-associated cardiovascular diseases, such as the uremic milieu and infection, have been explored [2]. More recently attention has been paid to the intestine and its microbiota as a potential source of inflammation due to changes in the gut microbial composition and disruption of the intestinal epithelial barrier structure and function in CKD. Levels of uremic toxins and their gut-derived precursors play an important role in the pro-inflammatory response and have been linked to changes in gut microbiota composition [3]. For a long time, the number of identified colon-derived solutes in humans remained limited. Recently, metabolome studies made an important contribution to the identification of colon-derived solutes by comparing the urinary metabolome from controls to that of patients with colectomy [4,5]. In addition, the comparison of the plasma metabolome from controls and hemodialysis patients revealed colon-derived solutes that accumulate when the kidneys fail. Among the identified colon-derived uremic solutes are the known uremic toxins p-cresyl sulfate (pCS), p-cresyl glucuronide (pCG), indoxyl sulfate (IxS), indole-3-acetic acid (IAA) and trimethylamine N-oxide (TMAO). Levels of all five solutes have previously been shown to be increased in patients with CKD [6,7,8,9]. Several biological effects of these compounds have been demonstrated, mainly affecting pathways playing a role in inflammation, metabolic function, cardiovascular disease and fibrosis [10]. More recently, alterations in the sulfur metabolism, prominent in CKD, have been linked to changes in the microbiota composition and function and should be taken into account when evaluating the potential role of uremic toxins and their precursors in immune response and inflammation at their side of origin [11].

## 2. The Gut Immune Homeostasis

A large number of microbiota reside in and on our body, with the largest microbial community located in the gastrointestinal tract [12]. All these microbiota exert metabolic functions, generating metabolites which directly influence our immune and nervous system and several distal organs via the circulation [13]. The human immune system plays an essential role in maintaining homeostasis with resident microbial communities, while resident microbiota per se shape the human immunity [14]. Most knowledge has been gained from studies with germ-free mice and colonized mice revealing the role of microbial colonization in the formation of lymphoid tissues and subsequent immune system development [15].

An important role is reserved for the intestinal epithelium which serves as an immunological guard forming a barrier between the intestinal lumen, containing microbiota and their derivatives and metabolites, and the internal milieu. The gastrointestinal barrier consists of intestinal epithelial cells with, at the apical side, tight junction proteins sealing the cellular layer and preventing translocation from the luminal side towards the blood side. After microbial stimulation, the epithelial cells express patterns of recognition receptors and produce chemotactic factors affecting both myeloid and lymphoid cells. Innate lymphoid cells in the submucosa can be stimulated to produce cytokines, while the dendritic cells in the submucosa often determine whether the response will be pro- or anti-inflammatory. The composition of the intestinal microbiota and their metabolic activity affects the immune response in the submucosa mainly via passive and active transcellular transport of solutes regulated at the apical membrane of the epithelial cells [13,16].

## 3. Gut Dysbiosis in CKD

The gut microbial composition is altered in CKD patients. This was initially observed in culture-based approaches observing increased counts of aerobes and decreased counts of anaerobes in fecal samples [17] and increased counts of aerobes and anaerobes in the small intestines of non-dialyzed and dialyzed patients with end-stage kidney disease (ESKD) compared to controls [18,19]. Metagenomic sequencing and qPCR confirmed the altered gut microbial composition in CKD, revealing a difference of 190 microbial operational taxonomic units (OTUs) between ESKD patients and healthy controls [3] and a decline of the short-chain fatty acid (SCFA)-generating bacteria, *Bifidobacterium* spp. and *Lactobacillus* spp., and an elevation of *Enterobacteriaceae* and *Escherichia coli* with progressive stages of CKD [20]. Recently, Wang et al. demonstrated that alterations in the gut microbiome mediate metabolome changes in patients with ESKD [21]. The alterations in the gut microbial composition, as a potential cause of a change in the gut biochemical milieu in CKD, is further enhanced by several uremia-related factors, while the gut microbiota per se also contribute to CKD by generating the uremic toxin precursor metabolites. This indicates that there is a bidirectional relationship between the gut microbiota and the kidneys, also known as the gut-kidney axis.

Gut dysbiosis contributes to the disruption of the intestinal barrier integrity causing translocation of bacteria and bacterial components which trigger the intestinal immune system. Under these conditions, intestinal epithelial cells will express pro-inflammatory cytokines, promoting a Th1 and Th17 response by dendritic cells and macrophages and producing increased levels of commensal-specific IgG by B cells. Binding of lipopolysaccharide (LPS) to its receptor complex on macrophages further results in enhanced production of pro-inflammatory cytokines [22]. Circulating endotoxin is a potential cause of inflammation in CKD [23,24,25]. A dysregulated immune response and chronic production of pro-inflammatory cytokines lead to systemic inflammation, which can further accelerate the progression of CKD and the development of CKD-associated cardiovascular disease.

## 4. End Metabolites of Intestinal Bacterial Metabolism

**Figure 1:**
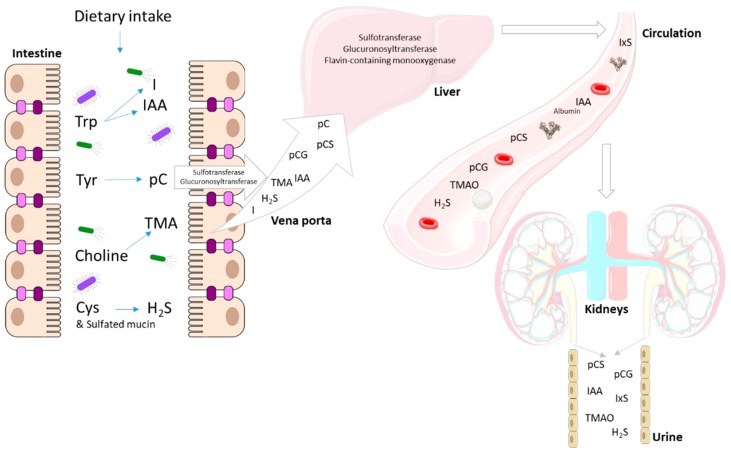
End products of the intestinal bacterial metabolism (I, IAA, pC, TMA, H_2_S) are transported as such or after detoxification (sulfatation and glucuronidation) towards the liver via the portal vein where the remaining pC and I are conjugated and TMA is oxygenated. The end products of the bacterial and human metabolism are taken up into the circulation (IxS, IAA, pCS, pCG, TMAO, H_2_S) where some bind to albumin (IxS, IAA, pCS and to a lesser extend pCG). H_2_S is also generated by different tissues throughout the body. Finally, these compounds are excreted into the urine. Trp: tryptophan; Tyr: tyrosine; Cys: cysteine; I: indole; IAA: indole-3-acetic acid; pC: p-cresol; TMA: trimethylamine; TMAO: trimethylamine N-oxide; pCS: p-cresyl sulfate; pCG: p-cresyl glucuronide; IxS: indoxyl sulfate.

The bacterial generation of uremic toxin precursors depends on the availability of carbohydrates at the level of the colon. In the presence of an excess of carbohydrates, amino nitrogen is mainly built into the growing bacterial biomass and saccharolytic fermentation prevails, producing SCFAs, keeping the pH low and suppressing the proteolytic activity [15,26]. However, in CKD, characterized by a decreased absorption of amino acids and abnormal intestinal motility in the small intestine and by a prolonged colonic transit [19,27], concentrations of amino acids are higher than expected in the colon. This induces an upstream expansion of proteolytic species and could lead to an increase in the metabolization of aromatic amino acids into precursors of uremic toxins, such as p-cresol, indole and trimethylamine (TMA), and of IAA (Figure 1). In addition, sulfur-containing dietary products and sulfated mucins are metabolized to form hydrogen sulfide (H_2_S) [11]. Currently, few reports on intestinal levels of these metabolites are available [28], while the existing data diverge [29,30]. In the following paragraphs, the potential biological effects of these metabolites at their side of origin are described, focusing on cells of the immune system and immune response, as summarized in Table 1.

### 4.1. p-Cresol

p-Cresol (108.14 g/mol) is an end product of the bacterial metabolization of the aromatic amino acid tyrosine (181.19 g/mol) in the colon. Kawakami et al. reported that fasting in rats enhances the production of p-cresol due to an increased concentration of endogenous protein in the caecum [31]. At the side of the colonic epithelial cells, in HT29 Glc(−/+) cells, p-cresol has been shown to significantly increase lactate dehydrogenase leakage and decrease adenosine triphosphate (ATP) content, while in Caco-2 cell monolayers, p-cresol significantly decreases the transepithelial electrical resistance and increases the paracellular transport of fluorescent isothiocyanate (FITC)-dextran [32]. In addition, the genotoxicity of p-cresol was suggested by the induction of DNA damage, affecting cell cycle kinetics in HT29 and Caco-2 cells [33]. These studies point to the local contribution of p-cresol to the disruption of the intestinal barrier function, which will indirectly affect the host immune response. Furthermore, incubation of *Lactobacillus casei Shirota*-stimulated J774.1 cells, a murine macrophage-like cell line, in the presence of p-cresol, inhibited IL-12 p40 production in a dose-dependent manner [34]. Previously, p-cresol was also shown to suppress monocyte and granulocyte respiratory burst activity [35]. These data suggest that at the side of the intestine, p-cresol could exert deleterious effects on the host defense system per se.

### 4.2. Indole and Indole-3-Acetic Acid

Both indole (117.15 g/mol) and indole-3-acetic acid (175.18 g/mol) are metabolites from the bacterial tryptophan (204.22 g/mol) metabolism. These metabolites are bioactive and can affect the intestinal barrier function and immune cells via the pregnane X receptor (PXR) and the aryl hydrocarbon receptor (AhR), both playing a role in the expression of the mucin 2 gene, which is essential for an intact epithelial barrier [36,37]. Indole functions as a signaling molecule in the gut homeostasis where it can regulate bacterial motility, biofilm formation, antibiotic resistance, and secretion of virulence factors affecting host cell invasion by non-indole-producing species, such as *Salmonella enterica* and *Pseudomonas aeruginosa* and even the yeast *Candida albicans* [38]. Indole has also been recognized as a beneficial signal molecule in intestinal epithelial cells. It ameliorates intestinal inflammation as demonstrated by the decreased TNF-α-mediated activation of nuclear factor (NF)-κB, the decreased expression of the pro-inflammatory chemokine IL-8, and the decreased attachment of pathogenic *E. coli* to the human enterocyte cell line, HCT-8 cells, as well as the increased expression of the anti-inflammatory cytokine IL-10 [39]. Indole also increased the expression of genes involved in strengthening the mucosal barrier and mucin production, which were consistent with an increase in the transepithelial resistance of HCT-8 cells [39].

Data on the local effect of IAA in the intestine are limited. Hendrikx et al. reported that restoration of intestinal levels of IAA protected mice from ethanol-induced steatohepatitis by inducing intestinal expression of IL-22 and antimicrobial C-type lectin regenerating islet-derived 3 gamma (REG3G), preventing translocation of bacteria to the liver [40].

In contrast to p-cresol, both indole and IAA exert beneficial effects in the gut by regulating gut homeostasis and by strengthening the mucosal barrier and mucin production.

### 4.3. Trimethylamine

The fishy, odorous trimethylamine (59.11 g/mol) is produced from the bacterial metabolization of dietary products such as phosphatidylcholine and carnitine, found in fish, seafood, eggs, cheese and red meat. TMA is rapidly absorbed through the gut wall and transported to the liver, where it is converted into nonodorous TMAO by the enzyme flavin-containing monooxygenase-3. Although no local intestinal toxic effects could be retrieved from literature, TMA was suggested to affect other barrier functions such as in the respiratory tract and the skin [41,42].

### 4.4. Sulfur Compounds

Among the sulfur compounds present in the gut, H_2_S is currently one of the most extensively studied. H_2_S (34.08 g/mol) is a gas produced by the body, with many biological functions among which anti-inflammatory and anti-oxidant properties. However, it must be kept in mind that H_2_S is also a lethal toxin when present at high concentrations, inhibiting complex IV in the mitochondrial respiratory electron transfer chain. 

The various forms in which H_2_S is present are: free H_2_S (gaseous H_2_S, along with the HS-hydrosulfide anion, and S^2−^ sulfide anion), the acid-labile sulfur (iron-sulfur clusters and persulfides), and the bound sulfane sulfur (thiol sulfides, polysulfides, sulfate/sulfite, and bound elemental sulfur). It is produced both enzymatically and nonenzymatically. Three enzymes are involved: cystathionine β-synthase (CBS, brain), cystathionine γ-lyase (CSE, vascular tissues, liver, kidney), and 3-mercaptopyruvate sulfurtransferase (MST, kidney). CBS and CSE belong to the transsulfuration pathway, which is mainly devoted to (1) the endogenous generation of cysteine, (2) homocysteine detoxification, and (3) H_2_S generation (and concomitant lanthionine production). In red blood cells (and other tissues, such as spleen, heart, lung, muscle, bone marrow) it is likely most often produced nonenzymatically, utilizing cysteine [43]. H_2_S can signal through four mechanisms [44]: (1) reduction and/or direct binding of metalloprotein heme centers; (2) antioxidant action through reactive oxygen species (ROS)/reactive nitrogen species scavenging; (3) post-translational modification of proteins by addition of a thiol (-SH) group onto reactive cysteine residues, so-called persulfidation (or S-sulfhydration), and formation of polysulfides [45,46]; (4) a postulated redox-linked metabolic reprogramming mechanism through sulfide quinone oxidoreductase in mitochondria [47].

Even considering the difficulties in accurately measuring H_2_S and the various species in which it is present [48], its concentration in tissues and blood is usually in the low micromolar range; however, in the luminal content of the large intestine the total sulfide concentration is about 0.2–2.4 mM [49]. The sources of H_2_S in the gut are the Sulfate Reducing Bacteria (SRB), gut fermentative bacteria, and colonic tissues. About 50% of H_2_S originates from the bacterial metabolization of sulfur-containing dietary products and sulfated mucins present in the outer mucus layer of the colon [50]. Colon epithelial cells are adapted to the high environmental H_2_S exposure; in fact, these cells possess an efficient mitochondrial H_2_S oxidation pathway, dedicated to its disposal. Intestinal epithelial cells express enzyme systems that efficiently degrade sulfide into thiosulfate and sulfate, which are absorbed and finally excreted in the urine [51,52]. 

To date, it is not entirely clear if gut H_2_S is a mediator of a productive mutualistic relationship at the host–microbial interface or a simple metabolic byproduct of enzymatic reactions, but this is less likely [42]. It is known that H_2_S is useful to the local gut bacteria because it increases bacterial resistance to antibiotics [53], it acts as a protective mechanism against ROS [54], and it provides bacterial resistance towards elimination by the host immune system [55]. However, the physiologic and regulatory roles of H_2_S in a healthy and a diseased gut are still not well defined. Mercaptides, such as H_2_S, help to maintain anaerobic conditions in the colonic lumen. There is even evidence that the recycling of H_2_S by a “thiosulfate shunt” integrates the enzymatic activities of both the intestinal epithelial cells and intestinal microbiota. On the other hand, high H_2_S levels have been shown to inhibit butyrate oxidation, an important energy source of the colonic epithelial cells [56]. In the end, H_2_S has beneficial effects on the local intestinal microbiota, but it could negatively affect the intestinal barrier function because it has been suggested that H_2_S plays a potential role in the etiology of bowel disorders such as inflammatory bowel diseases. In addition, gut-derived H_2_S is able to exert hypotensive effects [49]. 

It has been shown in a germ-free mice model that free H_2_S and bound sulfane sulfur are significantly reduced in plasma, while acid-labile sulfide levels remain normal [57]. In the same paper, CSE activity is also lower in several tissues, such as the aorta, the brain, and kidneys. H_2_S is also significantly lower in plasma from CKD and hemodialysis patients compared to controls, while the metabolic related compounds cystathione, homolanthionine and lanthionine are significantly increased [58]. In this respect, lanthionine, a byproduct of the transsulfuration pathway found to be quite high in the plasma of CKD patients, is able to exert several toxic effects [59,60,61,62]. Lanthionine is also an end product of bacterial metabolism. Very little is currently known about the levels of these compounds in the intestinal environment of CKD patients and whether differences in the gut microbiota composition present in CKD affect H_2_S levels or its effects.

## 5. Immunomodulatory Effects of Uremic Toxins from Colonic Origin

### 5.1. p-Cresyl Sulfate

Detoxification of p-cresol occurs in the mucosa of the colon [63] and in the liver [64], where it is sulfated into pCS (188.2 g/mol) and a small fraction is glucuronidated into pCG (283.3 g/mol) [65,66]. When entering the circulation these solutes bind to plasma albumin in a reversible manner [67]. pCS was suggested to cause immune dysfunction and increase the risk of infectious diseases in CKD patients. Schepers et al. demonstrated for the first time an in vitro biological effect of pCS at uremic concentrations, revealing an increase in the percentage of leukocytes displaying oxidative burst activity at baseline and an inhibition of the leukocyte burst activity after stimulation [68]. A synergistic activating effect was observed when the glucuronide conjugate of p-cresol, pCG, was added [69]. This was confirmed in an in vivo microscopic rat model where superfusion of the peritoneal membrane with pCS caused a rapid increase in the number of rolling leukocytes while the combination of both pCS and pCG caused impaired blood flow and vascular leakage but did not further enhance leukocyte rolling over pCS alone [70]. Activation of leukocytes per se and increased crosstalk between leukocytes and the vascular endothelial cells in the presence of pCS may contribute to the pro-inflammatory condition which is characteristic for CKD patients. In addition, Han and coworkers demonstrated that pCS promotes plaque growth and instability in ApoE KO mice by enhancing leukocyte-endothelium interaction [71].

Although the total uremic milieu was shown to suppress monocytic CYP27B1 expression, an enzyme regulating local production of 1,25-dihydroxyvitamin D3 in monocytes playing a role in the immunomodulatory effects of vitamin D, no specific contribution of pCS could be demonstrated [72]. Shiba et al. demonstrated that mice fed a tyrosine-rich diet causing pCS accumulation in their blood revealed a suppressed Th1-type cellular immune response demonstrated by the suppressed production of interferon (IFN)-gamma and the decreased percentage of IFN-gamma-producing Th1 cells in the presence of pCS [73]. Later on, the same group also demonstrated that pCS suppresses LPS-induced anti-bacterial immune responses in murine macrophages [74] and decreases peripheral B cells by inhibiting proliferation of CD43(+) B-cell progenitors in vitro [75]. pCS was shown to induce macrophage activation demonstrated by increased oxidative burst activity and phagocytosis but interfered in antigen processing, leading to a failure in adaptive immune response in CKD [76]. Levels of terminally differentiated CD8+ T cells also positively correlated with the level of pCS pointing to a premature aging phenotype of the immune system in patients with ESKD [77]. More recently, Borges Bonan and coworkers demonstrated that a combination of sulfated uremic toxins, among which pCS and IxS contribute to the increasing proportion of intermediate (CD14++CD16+) pro-inflammatory monocytes in CKD patients [78].

### 5.2. Indoxyl Sulfate

The intestinally generated indole is further metabolized in the liver to indoxyl sulfate (213.21 g/mol). Among other uremic toxins, IxS is a ligand of the AhR. The AhR regulates both innate and adaptive immune responses and is expressed in dendritic cells (DCs). The AhR inhibits the development of plasmacytoid DCs from bone marrow precursors induced by FMS-like tyrosine kinase-3 ligand (Flt3L), probably via inhibition of signal transducer and activator of transcription-3 (STAT3) expression [79]. The study by Ghimire et al. suggested an anti-inflammatory and tolerizing effect of IxS on human DCs [80]. However, in monocytes, the response to IxS through the AhR increases levels of TNF-α and upon stimulation with TNF-α, human vascular endothelial cells express CX3CL1, a chemokine ligand of CX3CR1 [81]. In addition, IxS induces monocytic ROS production in an NADPH oxidase-dependent manner [82]. The IxS-induced TNF-α production in macrophages is regulated through a mechanism involving the interaction of AhR, NF-κB, and the suppression of cytokine signaling [83]. IxS also increases LPS-induced NF-κB nuclear translocation, ROS release and altered calcium concentrations in J774A.1 macrophages, mainly because of mitochondrial calcium overloading [84]. In RAW 264.7 culture, NF-κB mRNA expression was stimulated by IxS, while nuclear factor erythroid 2-related factor 2 (Nrf2) was downregulated, triggering inflammation and oxidative stress [85]. Finally, IxS enhances leukocyte adhesion to the endothelial cells in vitro by the ROS/p38 MAPK pathway and the ROS/JNK/NF-κB pathway [82,86]. These effects indicate that IxS-mediated immune dysfunction may cause vascular endothelial cell damage in CKD patients.

### 5.3. Indole-3-Acetic Acid

In humans, indole acetic-3-acid directly originates from tryptophan metabolism by intestinal bacteria and from tryptophan in various tissues. The toxic effect of IAA in neutrophils is associated with cell peroxidase activity [87] and these processes have been involved in the activation of the glucose and glutamine metabolism [88]. There is a direct correlation between the cytotoxic effect of IAA and the peroxidase activity of the cells, with neutrophils presenting a higher peroxidase activity than inflammatory macrophages, whereas in resident macrophages and in lymphocytes this enzyme activity is very low [89]. IAA leads to marked structural changes and death of cultured neutrophils [87,89], whereas the toxic effects of IAA on lymphocytes are only observed when exogenous peroxidase is added to the culture medium or when they are co-cultivated with neutrophils [90]. In addition, IAA was shown to enhance glucose and glutamine metabolism in neutrophils and thioglycollate-elicited macrophages. IAA caused a marked increase in oxygen consumption by neutrophils, which was more pronounced in the presence of glutamine as compared to glucose. The stimulation of oxygen consumption leads to a reduction in the NADH/NAD+ ratio that activates the flux of substrates through the Krebs cycle [88]. Subcutaneous IAA administration in rats promotes an increased neutrophil phagocytic capacity without a pro-oxidant effect [91]. Finally, IAA was shown to damage DNA in human neutrophils. This genotoxicity negatively correlates with the antioxidant activities, as measured by the 2,2-diphenyl-1-picrylhydrazyl (DPPH) assay [92].

### 5.4. Trimethylamine N-Oxide

Flavin-containing monooxygenase enzymes encoded by the FMO gene family are involved in TMAO (75.11 g/mol) production from TMA in the liver, kidney and other tissues [93]. TMAO up-regulates vascular cell adhesion molecule-1 (VCAM-1) expression, promotes monocyte adherence and activates protein kinase C (PKC) and p-NF-κB. Interestingly, a PKC inhibitor was able to diminish the TMAO-stimulated VCAM-1 expression and monocyte adherence. Pharmacological inhibition revealed that endothelial cell adhesion of leukocytes was necessary for TMAO to induce inflammatory gene expression [94]. This points to the fact that TMAO promotes an early pathological process of atherosclerosis [95]. Haghikia et al. suggested that TMAO-related increase of pro-inflammatory monocytes may add to the elevated cardiovascular risk of patients with increased TMAO levels [96].

### 5.5. Sulfur Compounds

The effect of H_2_S on immune function has been investigated in different models, at different concentrations, with diverged results. H_2_S is generated at site of inflammation and can influence the ability of neutrophils to cause tissue injury. The H_2_S donor, sodium hydrosulfide, was shown to suppress leukocyte adherence to the vascular endothelium and to reduce leukocyte infiltration. Suppression of endogenous H_2_S synthesis through blockade of CSE abolished this anti-inflammatory effect [97]. Zanardo et al. demonstrated in an intra-vital microscopic rat model that H_2_S donors inhibited aspirin-induced leukocyte adherence in mesenteric venules, likely via the activation of ATP-sensitive K+ channels [98]. Inhibition of endogenous H_2_S synthesis elicited leukocyte adherence and leukocyte infiltration was also suppressed by H_2_S donors and exacerbated by inhibition of endogenous H_2_S synthesis [98]. These results suggest that endogenous H_2_S is an important mediator of acute inflammation, acting at the leukocyte-endothelium interface. Key discoveries concerning H_2_S were recently reviewed by Szabo [99]. 

H_2_S appears to play an important role in the modulation of the immune response by regulating the post-translational modification of the NF-kB pathway. However, depending on the model and the concentration used, H_2_S functions as an activator and an inhibitor towards this pathway [100]. H_2_S donors also inhibit macrophage pro-inflammatory cytokine production, as well as cyclooxygenase-2 and nitric oxide production, and decrease macrophage motility. CBS and CSE are regulated by ox-LDLs, while H_2_S mitigates ox-LDL-induced cholesterol efflux and foam cell formation [44]. Endogenous levels of sulfide reversibly inhibit the activity of circulating and endothelium-bound myeloperoxidase (MPO), suggesting a mediatory role on the oxidant-producing function of the enzyme. Furthermore, the produced polysulfides, together with MPO-catalyzed sulfide oxidation and the lack of interaction between MPO and sulfide oxidation products, may envisage a modulatory role of MPO in sulfide signaling [101]. It has been demonstrated that H_2_S is able to reduce inflammation in a mononuclear cell adhesion model by a mechanism related to ADAM17-dependent TNF-α activation [102]. Most studies on H_2_S and immune function are related to cellular immunity. However, it has been shown that antibody cleavage of the disulfide bonds between heavy and light chains and the ensuing sulfhydration is induced by H_2_S donors, which also suppresses the alternative complement activation pathway. H_2_S-treated antibodies exhibit a marked reduction in antigen-binding ability, and H_2_S inhibits cell lysis in glomerular mesangial cells and in human T lymphocytes [103]. 

Little is known about this topic in CKD and dialysis patients. In uremia, CSE activity is reduced in blood mononuclear cells from uremic patients on hemodialysis [104]. CSE downregulation could be due to a uremic toxin, perhaps lanthionine, which is able to reduce H_2_S production in hepatoma cells. It is currently unknown if the gut microbiome is able to influence immune function in CKD patients through sulfur compounds and in particular through H_2_S. 

## 6. Conclusions

Several uremic metabolites and their precursors exert immunomodulatory effects both at their side of origin and especially in the circulation. At the side of the intestine p-cresol, TMA and H_2_S can affect the intestinal barrier structure and function and could in this way stimulate cells of the immune system and contribute to inflammation. In contrast, the bacterial metabolization products of tryptophan, indole and IAA, seem to be beneficial for the intestinal epithelial cells and the intestinal mucosa. Aiming at a decreased generation of especially p-cresol, by e.g., changing dietary intake [105] or by the administration of pre- [106], pro- [107] or synbiotics [108], could contribute to the intactness of the intestinal barrier function, avoiding pro-inflammatory reactions. However, in view of the local intestinal beneficial effects of end products of the bacterial tryptophan metabolism, intestinal absorption, by affecting transporter protein expression and/or function, rather than generation, should be explored and targeted. Both strategies will contribute to decreased levels of circulating protein-bound uremic toxins and might reduce micro-inflammation in CKD. To date, little is known about the role of gut sulfur compounds and H_2_S, in particular in the context of CKD, where a profound dysregulation of their metabolism is present.

## Figures and Tables

**Table 1:** (Patho)physiological effects of intestinally generated uremic toxins and their precursors at their side of origin.

Metabolites	Side of Origin	(Patho) Physiological Effects	Ref.
**Metabolites Generated by Gut Bacteria**			
p-Cresol	Colon	*Intestinal epithelial cells:*	
		↑LDH leakage	[32]
		↓ATP content	
		↓TEER	
		↑Paracellular transport	
		Genotoxicity	[33]
		*Leukocytes/Macrophages:*	
		↓IL-12 p40 production	[34]
		↓Respiratory burst activity	[35]
Indole	Colon	*Intestinal epithelial cells:*	
		↑Mucin2 expression	[36,37]
		Regulation of gut homeostasis	[38]
		↓TNF-α mediated NF-κB activation	[39]
		↓IL-8 expression	
		↑IL-10 expression	
		↑TER	
Indole-3-acetic acid	Colon	*Intestinal epithelial cells:*	
		↑IL-22 expression	[40]
		↑Antimicrobial C-type lectin REG3G	
Trimethylamine	Colon	ND	
H_2_S	Colon	*Intestinal bacteria:*	
		↑Antibiotic resistance	[53]
		Protection against ROS	[54]
		Protection against immune cells	[55]
		Maintenance of anaerobic conditions	
		↓Butyrate oxidation	[56]
**Circulating Uremic Toxins**			
p-Cresyl sulfate	Intestinal epithelial cells and liver	*Leukocytes:*	
		↑Baseline ROS and ↓ROS after stimulation	[68]
		↑Rolling	[70]
		↑Plaque growth and instability	[71]
		↓IFNγ-producing Th1 cells	[73]
		↓Anti-bacterial immune response	[74]
		↓Proliferation of CD43(+) B cell progenitors	[75]
		↑Macrophage activation	
		↓Antigen processing	[76]
		Premature aging of immune cells	[77]
p-Cresyl glucuronide	Intestinal epithelial cells and liver	*Leukocytes:*	
		Synergistic to pCS: ↑ROS, impaired blood	[69]
		flow; vascular leakage	[70]
Indoxyl sulfate	Liver	Anti-inflammatory and tolerizing effect on	[80]
		DCs	
		*Monocytes:*	
		↑TNF-α→↑HUVEC CX3CL1	[81]
		↑ROS	[82]
		↑Leukocyte-endothelial cell adhesion	[82,86]
		*Macrophages:*	
		↑TNF-α	[83]
		↑NF-κB, ROS, mitochondrial Ca^2+^	[84]
		overload	
		↓Nrf2	[85]
Indole-3-acetic acid	Colon	*Neutrophils:*	
		↑peroxidase activity	[87]
		↑Glucose and glutamine metabolism	[88]
		↑Oxygen consumption	[88]
		↑Structural changes and cell death	[87,89]
		↑Phagocytic activity	[91]
		Genotoxicity	[92]
Trimethylamine-oxide	Liver, kidney, and other	↑VCAM-1	[94]
		↑Monocyte-endothelial	
		adhesion→↑inflammatory gene expression	
		Activates PKC and p-NF-κB	
H_2_S	Brain, vascular tissue, liver, kidney, RBC, and other	*Leukocytes:*	
		↓Leukocyte-endothelial adhesion	[97,98]
		↓Leukocyte infiltration	
		Regulation of post-translational	[100]
		modification of NF-κB pathway	
		*Macrophages:*	
		↓Pro-inflammatory cytokine production	[44]
		↓COX-2 and NO production	
		↓Macrophage motitlity	
		↓MPO activity	[101]
		↓Inflammation	[102]
		↓Antigen-binding	[103]
		↓Cell lysis (glomerular mesangial cells and T-lymphocytes)	

LDH: lactate dehydrogenase; ATP: adenosine triphosphate; TE(E)R: transepithelial (electrical) resistance; IL: interleukin; REG3G: regenerating islet-derived 3 gamma;ND: none described; RBC: red blood cells: ROS: reactive oxygen species; DCs: dendritic cells; HUVEC: human vascular endothelial cells; VCAM: vascular cell adhesion molecule: PKC: protein kinase C; COX: cyclooxygenase; NO: nitric oxide.; MPO: myeloperoxidase. ↓: decreased; ↑: increased.
